# An embedded randomised controlled retention trial of personalised text messages compared to non-personalised text messages in an orthopaedic setting

**DOI:** 10.12688/f1000research.24244.1

**Published:** 2020-06-11

**Authors:** Alex S. Mitchell, Liz Cook, Alexandra Dean, Caroline Fairhurst, Matthew Northgraves, David J. Torgerson, Mike Reed

**Affiliations:** 1 Department of Health Sciences, University of York, UK, York, Y010 5DD, UK; 2 Hull Health Trials Unit, University of Hull, Hull, HU6 7RU, UK; 3 North Tyneside General Hospital, Northumbria Healthcare NHS Foundation Trust, North Shields, Tyne and Wear, NE29 8NH, UK

**Keywords:** SWAT, Study Within A Trial, attrition, SMS, text messages

## Abstract

**Background:** Several studies have investigated whether personalising trial documentation can aid recruitment and retention. We did a ‘study within a trial’ (SWAT) evaluating the effectiveness of a personalised text message compared to a non-personalised text message, on the retention rate in a large orthopaedic trial.

**Methods: **The SWAT was embedded in the Knee Replacement Bandaging Study (KReBS) trial. The primary outcome was the proportion of 12-month questionnaires returned. Secondary outcomes were the proportion of questionnaires completed and time to questionnaire return. Binary data were analysed using logistic regression and time to return using Cox proportional hazards regression.  Odds ratios (OR) and hazard ratios (HR) are presented, with associated 95% confidence intervals (CI) and p-values.

**Results:** In total, 1465 participants were included in the SWAT. In the personalised group, 644/723 (89.1%) of participants returned a questionnaire, compared to 654/742 (88.1%) in the non-personalised group. The absolute difference in return rate was 0.9% (95% CI: -2.3% to 4.2%; p=0.57). There was no evidence of a difference between the groups in the likelihood of returning a questionnaire (OR 1.09; 95% CI: 0.79 to 1.51; p=0.61), the likelihood of returning a complete questionnaire (OR 1.11; 95% CI: 0.82 to 1.51; p=0.50) nor in time to return (HR 1.05; 95% CI: 0.94 to 1.17; p=0.40).

**Conclusion: **This SWAT adds to the growing evidence base for whether personalised text messages are effective.

**Registration:** ISRCTN87127065 (20/02/2017); SWAT 35 (01/12/2015)

## Introduction

Clinical trialists have identified the recruitment and retention of participants as key issues for randomised controlled trials (RCT) ^1, 2^.

Several studies have investigated whether personalising trial documentation can aid recruitment and retention ^3, 4^. Recently, Cochrane *et al*. looked at the effect of personalised text messages compared to standard text messages in improving retention rates ^5^. This study was carried out in response to a number of embedded trials evaluating the effectiveness of SMS messages in improving retention rates ^6– 11^, alongside a study suggesting personalised messages increased the payment of delinquent fines ^12^.

To further add to the evidence on the effectiveness of personalised text messages, we did a ‘study within a trial’ (SWAT) evaluating the effectiveness of a personalised text message compared to a non-personalised text message on postal questionnaire response rates in a large orthopaedic trial.

## Methods

### Design

This paper details the methods and results of a SWAT embedded within the prospectively registered Knee Replacement Bandaging Study (KReBS) RCT ( ISRCTN87127065, registered on 20 February 2017). KReBS evaluated the effectiveness of a two-layer compression bandage compared with a standard wool and crepe bandage applied post-operatively on patient-reported outcomes in total knee replacement patients ^13^.

### Participants

The SWAT was conducted in 26 NHS hospital trust sites and was implemented at the start of the study. All KReBS participants were eligible for this SWAT provided they had opted in to receiving SMS messages and were not deceased or withdrawn from follow-up before being due to be sent their 12-month postal questionnaire.

### Intervention

Participants in the SWAT were sent either a personalised or non-personalised text message ( Table 1) four days after their 12-month questionnaire was sent.

**Table 1.** Description of the contents of the personalised and non-personalised text messages.

Text message type	Text message content
Personalised	“KReBS Trial: [ *Title*] [ *Surname*] you should have received a questionnaire in the post by now. Your answers are important; so please help by returning it as soon as you can. Thanks”
Non-personalised	“KReBS Trial: You should have received a questionnaire in the post by now. Your answers are important; so please help by returning it as soon as you can. Thanks”

KReBS - Knee Replacement Bandaging Study

### Outcomes

The primary outcome was the proportion of participants who returned a 12-month questionnaire. Secondary outcomes were the proportion of participants who completed the questionnaire and time to questionnaire return. A questionnaire was considered complete if the participant had answered 11 or more questions of the 12-item host trial primary outcome, the Oxford Knee Score ^14^.

### Sample size

Since this was an embedded trial, the sample size was determined by the number of participants in the main KReBS trial ^13^, which aimed to recruit 2600 participants.

### Randomisation

Participants were randomised into the SWAT using simple randomisation in a 1:1 allocation ratio. The allocation schedule was generated by a researcher at the York Trials Unit not involved in the recruitment or follow-up of participants.

### Blinding

Participants were not informed of their explicit participation in the SWAT, but due to the nature of the intervention could not be blinded to whether the text was personalised or non-personalised. Similarly, it was not possible to blind research staff to SWAT allocation.

### Approvals

The SWAT was approved by the Research Ethics Committee North East – Newcastle and North Tyneside on 13/04/2018 (REC Number 16/NE/0400; Amendment Number 16/NE/0400/AM14). As the SWAT was deemed to be low risk, explicit informed consent was not obtained for participation.

## Statistical analysis

Analyses were carried out using Stata v16.0 ^15^. A diagram detailing the flow of participants through the SWAT is provided, and baseline characteristics are presented by SWAT allocation. Outcomes are summarised descriptively. Statistical tests were two-sided using a 5% significance level, and were done on an intention to treat basis. All analyses (except the calculation of the absolute difference in return rate which was estimated using the two-sample test of proportions) used mixed effects regression, adjusting for SWAT allocation and host trial allocation as fixed effects and trial site as a random effect. Relevant parameter estimates are presented with associated 95% confidence intervals and p-values.

The proportion of participants who returned a 12-month questionnaire, and proportion complete, was analysed using logistic regression. A second SWAT evaluating receipt of a pen on response rates was also embedded in KReBS at 12 months ^16^. In a sensitivity analysis, we additionally adjusted the primary model for pen SWAT allocation.

Time to questionnaire return was analysed using a Cox proportional hazards shared frailty model. Participants who did not return a questionnaire were censored at 90 days.

## Results

**Figure 1.**
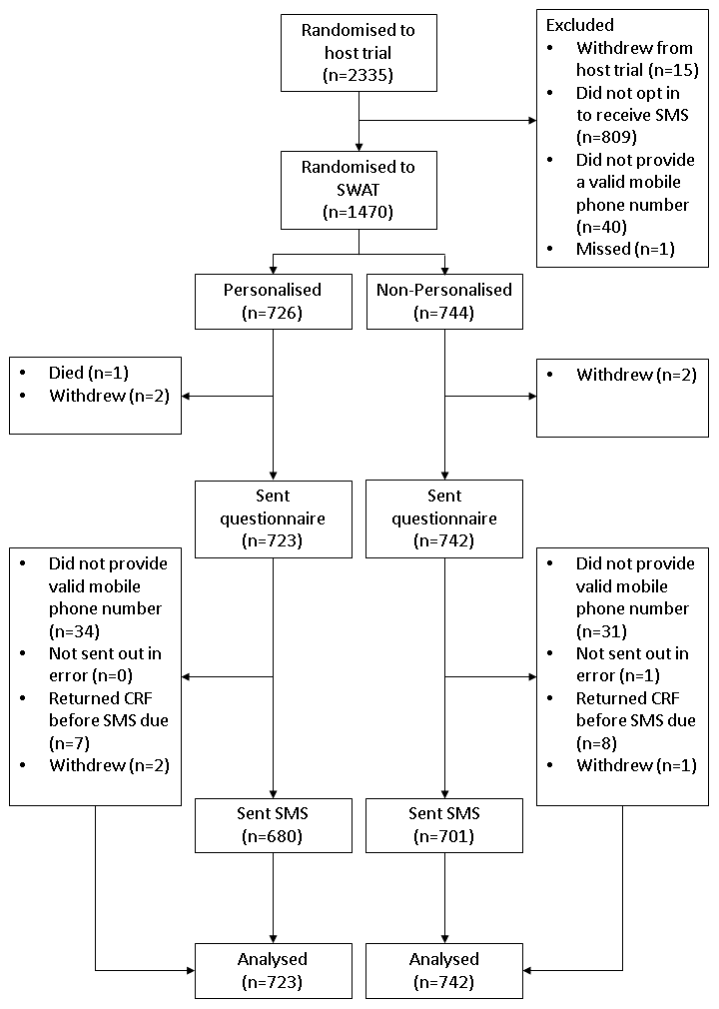
Study flow diagram.

In total, 2335 participants were recruited into the KReBS trial and 1470 were randomised to the SWAT ( Figure 1). The average age was 66.8 years and 54.0% were female ( Table 2 ^17^). Five participants died or withdrew following randomisation and as a result 723 participants in the personalised group, and 742 in the non-personalised group, were sent a 12-month questionnaire and were included in the analysis. Of these, 680 (94.1%) of the 723 participants in the personalised group, and 701 (94.5%) of the 742 in the non-personalised group, were sent a text.

**Table 2.** Baseline characteristics of the study within a trial (SWAT) participants.

	Personalised (n=726)	Non-personalised (n=744)	Total (n=1470)
**Gender, n (%)** Male Female Missing	335 (46.1) 391 (53.9) 0 (0)	340 (45.7) 403 (54.2) 1 (0.1)	675 (45.9) 794 (54.0) 1 (0.1)
**Age** n (%) Mean (SD) Median (IQR)	726 (100) 66.9 (8.5) 67.2 (60.7, 72.9)	743 (99.9) 66.8 (8.5) 67.0 (60.8, 72.4)	1469 (99.9) 66.8 (8.5) 67.1 (60.8, 72.7)
**Oxford Knee Score** n (%) Mean (SD) Median (IQR)	576 (79.3) 20.4 (8.0) 20 (14, 26)	582 (78.2) 20.5 (8.0) 20 (15, 26)	1158 (78.8) 20.4 (8.0) 20 (15, 26)

In the personalised group, 644/723 (89.1%) participants returned a questionnaire, compared to 654/742 (88.1%) in the non-personalised group ( Table 3 ^17^). The absolute difference in return rate was 0.9% (95% CI: -2.3% to 4.2%; p=0.57). There was no evidence of a difference between the groups in the likelihood of returning a questionnaire (OR 1.09; 95% CI: 0.79 to 1.51; p=0.61), the likelihood of returning a complete questionnaire (OR 1.11; 95% CI: 0.82 to 1.51; p=0.50) nor in time to return (HR 1.05; 95% CI: 0.94 to 1.17; p=0.40). In total, 1465 participants were also randomised to the pen SWAT. When the primary model was repeated with the addition of pen SWAT allocation, the results remained the same.

**Table 3.** Descriptive summaries of primary and secondary outcomes.

	Personalised (n=723)	Non-personalised (n=742)	Total (n=1465)
**Returned questionnaire, n (%)** Yes No	644 (89.1) 79 (10.9)	654 (88.1) 88 (11.9)	1298 (88.6) 167 (11.4)
**Completed questionnaire, n (%)** Yes No	634 (87.7) 89 (12.3)	641 (86.4) 101 (13.6)	1275 (87.0) 190 (13.0)
**Time to return, days** n (% Mean (SD) Median (IQR)	644 (100) 15.9 (15.0) 10 (8, 16)	654 (100) 17.0 (20.4) 10 (8, 16)	1298 (100) 16.5 (17.9) 10 (8, 16)

## Discussion

This embedded trial found little evidence to suggest personalised text messages are more effective than non-personalised text messages in encouraging return and completion of questionnaires. The trial did not find evidence of a statistically significant difference between groups in any of the outcomes, although effect size estimates favoured the personalised group. On the other hand, while Cochrane and colleagues also did not find evidence of a statistically significant difference between groups, estimates of effect mostly favoured the non-personalised group ^5^.

The SWAT had a large sample size, which means the results can be generalised to other orthopaedic studies. However, completion rate was calculated as a proportion of all SWAT participants rather than all SWAT participants who returned a questionnaire, and as a result questionnaire completion was highly correlated with questionnaire return. In addition, some participants included in the analysis did not receive a text message.

## Conclusion

This SWAT adds to the growing evidence base for whether personalised trial documentation, in particular text messages, are effective.

## Data availability

### Underlying data

Open Science Framework: Underlying data and CONSORT diagram for an embedded randomised controlled retention trial of personalised text messages compared to non-personalised text messages in an orthopaedic setting. https://doi.org/10.17605/OSF.IO/KHJ8E ^17^

This project contains the following underlying data:

- KReBS_Text_SWAT_Clean.sas (Study data in SAS compatible format)
- KReBS_Text_SWAT_Clean.csv (Study data in .csv format)
- KReBS_Text_SWAT_Clean_Key.xlsx (Key for datasets)

### Reporting guidelines

Open Science Framework: CONSORT checklist for ‘An embedded randomised controlled retention trial of personalised text messages compared to non-personalised text messages in an orthopaedic setting’ https://doi.org/10.17605/OSF.IO/KHJ8E ^17^

Data are available under the terms of the Creative Commons Zero "No rights reserved" data waiver (CC0 1.0 Public domain dedication).
