# Supplementary material for: An embedded randomised controlled retention trial of personalised text messages compared to non-personalised text messages in an orthopaedic setting
Source: F1000Res. 2021 Sep 15;9:591. Originally published 2020 Jun 11. [Version 2] doi: 10.12688/f1000research.24244.2 (PMC9091807; doi:10.12688/f1000research.24244.2)
Supplement: Peer review history

# Peer review: Reviewer response for version 2

**DOI:** 10.5256/f1000research.77319.r128647

**Published:** 2022-05-10

**Authors:** David J Keene

**Affiliations:** 1 University of Oxford, Oxford, UK

Thank you for the invitation to review this interesting SWAT report. I note the other peer reviewers comments and responses. Overall this concise summary documents a well-designed and conducted SWAT. The authors are to be commended given the delivery was within a large and complex clinical trial.

 My recommendations to improve the manuscript are as follows:

 Abstract – the context of the intervention, a ‘personalised text message’, was not clear in isolation in the abstract, i.e. it was sent 4 day after sending a 12-month postal questionnaire to prompt completion.

 Methods

 The details of the KReBS trial are signposted but it would be helpful to have more background for the reader e.g. country, dates of conduct, main eligibility criteria for the main trial.

 For the intervention, stating the aim of the personalised message would aid clarity, it appears to be a prompt rather than a reminder but would be good to be explicit about this.

 Randomisation – how was the allocation schedule generated, computer programme?

 The statistical approaches seem reasonable but this is not my area of expertise.

 Discussion

 Some interpretation of the results in the context of other trials that have used personalised vs non-personalised text messages for first time postal questionnaire completion would be informative. Other SWATs have looked at personalisation as a reminder SMS after no postal return rather than a proactive prompt. Differences in host trials may also be important, there were good response levels in this host trial overall.

 The 95% CI includes a small negative to positive effect on response rate (-2.3% to 4.2%). Would the authors see value of further SWATs to enable meta-analysis and improve precision in the estimates? With a low cost and simple intervention such as this, a marginal difference could be considered valuable.

Is the work clearly and accurately presented and does it cite the current literature?

Partly

If applicable, is the statistical analysis and its interpretation appropriate?

I cannot comment. A qualified statistician is required.

Are all the source data underlying the results available to ensure full reproducibility?

Yes

Is the study design appropriate and is the work technically sound?

Yes

Are the conclusions drawn adequately supported by the results?

Yes

Are sufficient details of methods and analysis provided to allow replication by others?

Yes

Reviewer Expertise:

Trauma, Orthopaedic and Rehabilitation Clinical Trials

I confirm that I have read this submission and believe that I have an appropriate level of expertise to confirm that it is of an acceptable scientific standard.

# Peer review: Reviewer response for version 1

**DOI:** 10.5256/f1000research.26744.r64613

**Published:** 2020-07-03

**Authors:** Michelle E. Kho

**Affiliations:** 1 School of Rehabilitation Science, McMaster University, Hamilton, ON, Canada

Mitchell et al. elegantly describe a SWAT of personalized vs. non-personalized reminder text messages embedded in a randomized orthopaedic trial. The primary outcome was questionnaire return at 12 months, and the secondary outcomes were proportion of completed questionnaires, and time to questionnaire return. There was no difference in any outcome between groups.

 Minor issues:

1. There is a large body of evidence outlining strategies to improve return rates of postal questionnaires ^1^. The primary outcome in this study, questionnaire return at 12 months, could be confounded by the host trial efforts to improve postal questionnaire return. Can the authors report more information on the host trial efforts to optimize postal questionnaire return?
2. The questionnaire return rates for this subgroup of host trial participants was very high (~89%). Can the authors report the questionnaire return rates for the 809 people in the host trial who were not included in the SWAT, or if this is not available, the overall questionnaire return rate for the host trial?
3. In Figure 1, can you please clarify the difference between those excluded in the first box for “did not provide a valid mobile phone number” and those who were sent a questionnaire and “did not provide a valid phone number”?

Is the work clearly and accurately presented and does it cite the current literature?

Yes

If applicable, is the statistical analysis and its interpretation appropriate?

Yes

Are all the source data underlying the results available to ensure full reproducibility?

Yes

Is the study design appropriate and is the work technically sound?

Yes

Are the conclusions drawn adequately supported by the results?

Yes

Are sufficient details of methods and analysis provided to allow replication by others?

Yes

Reviewer Expertise:

Research methodology, trial design and reporting

I confirm that I have read this submission and believe that I have an appropriate level of expertise to confirm that it is of an acceptable scientific standard.

## References

[1] : Methods to increase response to postal and electronic questionnaires. *Cochrane Database Syst Rev* .2009; 10.1002/14651858.MR000008.pub4 MR000008 10.1002/14651858.MR000008.pub4 19588449PMC8941848

# Response

**Published:** 2021-08-31

**Authors:** Alex Mitchell

**Affiliations:** Mr, UK

We thank the reviewer for her positive feedback and constructive comments, to which we have responded below. In addition to responding to the comments, we have made a minor amendment to the results of the paper, as since publication a duplicate randomisation was found, and therefore 2334 participants were randomised to the host trial, not 2335. This does not impact the results of the SWAT, as the duplicate randomisation was not given an allocation in the SWAT.

**There is a large body of evidence outlining strategies to improve return rates of postal questionnaires1. The primary outcome in this study, questionnaire return at 12 months, could be confounded by the host trial efforts to improve postal questionnaire return. Can the authors report more information on the host trial efforts to optimize postal questionnaire return?**

 Thank you, we have added text under the Intervention section of the paper, describing the strategies used in the host trial to optimise questionnaire return.

**The questionnaire return rates for this subgroup of host trial participants was very high (~89%). Can the authors report the questionnaire return rates for the 809 people in the host trial who were not included in the SWAT, or if this is not available, the overall questionnaire return rate for the host trial?**

 In total, 864 participants were not randomised to the SWAT. This number consists of the 806 who did not opt-in to receive SMS, and the 58 who had either withdrawn, did not provide a valid mobile phone number or were missed. Of these 864, 827 had not withdrawn by the 12-month time point and were sent a questionnaire. 704 returned the questionnaire, which corresponds to a response rate of 85.1%. This is slightly lower than the response rate observed in the SWAT, which may be due to participants who didn’t opt-in to receive SMS or didn’t provide a valid mobile phone number being less engaged with the study. Due to this being a post-hoc analysis, we have decided not to include this result in the publication.

**In Figure 1, can you please clarify the difference between those excluded in the first box for “did not provide a valid mobile phone number” and those who were sent a questionnaire and “did not provide a valid phone number”?**

 We have added text describing the two stages of mobile phone number verification in the Intervention section of the paper.
